# Draft Genome Sequence of Clostridium estertheticum-like Strain FP4, Isolated from Spoiled Uncooked Lamb

**DOI:** 10.1128/MRA.00338-20

**Published:** 2020-05-07

**Authors:** Nikola Palevich, Faith P. Palevich, Paul H. Maclean, Ruy Jauregui, Eric Altermann, John Mills, Gale Brightwell

**Affiliations:** a AgResearch Limited, Grasslands Research Centre, Palmerston North, New Zealand; b Riddet Institute, Massey University, Palmerston North, New Zealand; University of Maryland School of Medicine

## Abstract

In order to improve the phylogenetic resolution of the genus *Clostridium* and our limited knowledge of meat spoilage caused by Clostridium estertheticum, the genome of *C. estertheticum*-like strain FP4 was sequenced. Here, we describe the 4.1-Mb draft genome sequence of *C. estertheticum*-like strain FP4, isolated from vacuum-packaged refrigerated spoiled lamb.

## ANNOUNCEMENT

Clostridium estertheticum-like strain FP4 is a Gram-positive, spore-forming, and slow-growing psychrotrophic bacterium that requires strict anaerobic conditions to grow (1). Contamination with different *Clostridia* species has been recognized as a major cause of blown-pack spoilage (BPS) in vacuum-packed meat products associated with a variety of animal types, including cattle, sheep, and deer (2). Recent amplified rRNA gene restriction analysis (ARDRA) profiling and a positive real-time *C. estertheticum*-like-specific PCR assay (3, 4) placed strain FP4 into the nontoxigenic *C. estertheticum*-like cluster with >89% similarity. This paper presents the draft genome sequence of *C. estertheticum*-like strain FP4 to facilitate future investigations into meat spoilage caused by psychrotrophic *Clostridium* species.

Strain FP4 was originally isolated in 2017 at AgResearch Ltd., Palmerston North, New Zealand, from a slightly distended package of vacuum-packed refrigerated spoiled lamb in which the meat had no discoloration. FP4 was originally isolated and cultured anaerobically from the meat drip at 10°C in 10-fold suspensions of prereduced peptone-yeast extract-glucose-starch (PYGS) broth (4), and phenol-chloroform genomic DNA extraction was conducted in accordance with a previously described method (5). Paired-end (PE) sequencing libraries were generated with a TruSeq Nano DNA library prep kit (Illumina, San Diego, CA, USA), followed by whole-genome sequencing on the MiSeq platform with a MiSeq reagent kit v2 (2 × 250 bp). Trimmomatic v0.39 (http://www.usadellab.org/cms/?page=trimmomatic) was used to filter the raw reads (Q score, >15 in a sliding window of 4 nucleotides [nt]; minimum length, >36 nt), yielding a total of 4,534,616 PE raw sequences. *De novo* assembly was conducted with A5-miseq v20169825 (6). Default parameters were used for all software unless otherwise specified.

*In silico* analysis of FP4 yielded 251 scaffolds with an overall genome coverage of 242×, a total length of 4,093,822 bp, and a G+C content of 31.2%. The FP4 assembly produced an *N*_50_ value of 43,169 bp, with the largest scaffold being 133,780 bp long. The genome sequence of FP4 reported here is a useful resource for the future development of strategies for early identification of the presence of clostridial spoilage and reduction in the onset of BPS.

### Data availability.

The draft genome sequence of Clostridium estertheticum-like strain FP4 has been deposited in GenBank under accession number JAAMNG000000000 and BioProject accession number PRJNA574489. The raw sequence reads were deposited in the Sequence Read Archive (SRA) under accession number SRR11113220.
